# Development of a Multi-Study Repository to Support Research on Veteran Health: The VA Cooperative Studies Program Epidemiology Center-Durham (CSPEC-Durham) Data and Specimen Repository

**DOI:** 10.3389/fpubh.2021.612806

**Published:** 2021-02-17

**Authors:** Meghan C. O'Leary, R. Lawrence Whitley, Ashlyn Press, Dawn Provenzale, Christina D. Williams, Blair Chesnut, Rodney Jones, Thomas S. Redding, Kellie J. Sims

**Affiliations:** ^1^Cooperative Studies Program Epidemiology Center-Durham, Durham Veterans Affairs Health Care System, Durham, NC, United States; ^2^RTI International, Research Triangle Park, NC, United States; ^3^Duke University Medical Center, Durham, NC, United States; ^4^Duke Molecular Physiology Institute, School of Medicine, Duke University, Durham, NC, United States

**Keywords:** repository, data sharing, veteran, biospecimen, genomics

## Abstract

Federal agencies, including the Department of Veterans Affairs (VA), have prioritized improved access to scientific data and results collected through federally funded research. Our VA Cooperative Studies Program Epidemiology Center in Durham, North Carolina (CSPEC-Durham) assembled a repository of data and specimens collected through multiple studies on Veteran health issues to facilitate future research in these areas. We developed a single protocol, request process that includes scientific and ethical review of all applications, and a database architecture using metadata (common variable descriptors) to securely store and share data across diverse studies. In addition, we created a mechanism to allow data and specimens collected through older studies in which re-use was not addressed in the study protocol or consent forms to be shared if the future research is within the scope of the original consent. Our CSPEC-Durham Data and Specimen Repository currently includes research data, genomic data, and study specimens (e.g., DNA, blood) for three content areas: colorectal cancer, amyotrophic lateral sclerosis, and Gulf War research. The linking of the study specimens and research data can support additional genetic analyses and related research to improve Veterans' health.

## Introduction

Biobanking encompasses all procedures needed to collect, process, store, and share specimens collected from human subjects as well as the policies that govern these activities (1). Stored biospecimens along with linked clinical and research data can and have been used to advance translational and population health research and support personalized medicine within clinical care (1, 2). The development and maintenance of data and specimen repositories commonly involves substantial resources, including dedicated staff, laboratory space and equipment, creation of standard operating procedures or related protocols, information technology systems, and funding (3). In addition, many ethical considerations are involved in obtaining participants' informed consent to use and share their data and specimens through biobanks and other repositories; examples include determining the appropriate type of consent to use and ensuring participants understand all procedures and potential risks and benefits (1–4). Despite these challenges, prior research has shown that participants are generally willing to have their information shared for future research purposes (5, 6). Potential benefits of data and specimen sharing include increasing efficiency of limited research resources, minimizing the burden of research participants and potential risks of research participation, and contributing to more generalizable knowledge intended to improve patient health and care (7).

Among federal agencies, increased transparency of and access to federally funded research results and scientific data have been prioritized over the past decade. In February 2013, the White House's Office of Science and Technology Policy issued a memorandum requiring federal agencies to make the results of their research collected with federal funds publicly available to support future research and innovations (8). This directive also required agencies to make their scientific data available to the public to the extent possible (8). In response, the U.S. Department of Veterans Affairs (VA) issued guidance on increasing public access to research data while continuing to protect the privacy of its Veteran patients. Beginning in December 2015, VA researchers were required to submit written data management plans with their protocols outlining how the data would be made available and describing the mechanisms for ensuring privacy, confidentiality, and long-term preservation and storage of the data (9).

As of July 2020, the VA's Office of Research and Development (ORD) included data and specimen sharing within two of its three strategic priorities for VA research (10). These priorities include ensuring that research findings are translated into clinical applications that improve the care of Veterans, and facilitating larger-scale research that can benefit Veterans and the general public (10). Noted activities to achieve these goals include the curation of linked and standardized data sources and the collection of biospecimens for genomic analysis (10).

The VA Cooperative Studies Program Epidemiology Center located in Durham, North Carolina (CSPEC-Durham) is one of many research programs under VA ORD oversight (11). We aimed to develop a repository to enable data and specimen sharing that was consistent with VA and ORD guidance and priorities and that would support additional epidemiologic and genomic research specific to the health needs of Veterans.

In this paper, we describe our center's process for developing a repository of research data and biological specimens collected from Veterans with and without chronic disease for sharing with investigators with approved research protocols. The CSPEC-Durham Data and Specimen Repository, subsequently referred to as the CSPEC-Durham Repository, houses data and specimens collected from multiple research studies on diverse Veteran health issues for the purpose of facilitating future research intended to improve the health of Veterans. The purpose of this report is to describe: (1) the selection of studies included in this repository, (2) the design of metadata-driven architecture for securely storing and tracking data and specimens, and (3) the development of a process to review the scientific and ethical merit of data and specimen requests.

## Materials and Methods

### Identifying Feeder Studies and Potential Sharing Restrictions

We first identified all studies conducted by members of the CSPEC-Durham research team for possible inclusion in the repository. These studies were evaluated as potential feeder studies, defined as individual research studies with a protocol approved by an Institutional Review Board (IRB) for which the collected data and, if applicable, specimens would be stored and available for sharing through the CSPEC-Durham Repository. We considered active studies with data collection and analysis still in progress, as well as legacy studies for which data collection and analysis had already been completed. Each research study focused on Veteran health issues and enrolled all or predominantly Veteran participants. We included studies that addressed different types of chronic disease areas affecting Veterans, as well as studies that enrolled Veterans with or without a particular illness to support research on risk factors, early detection, and progression of these illnesses.

**Figure 1:**
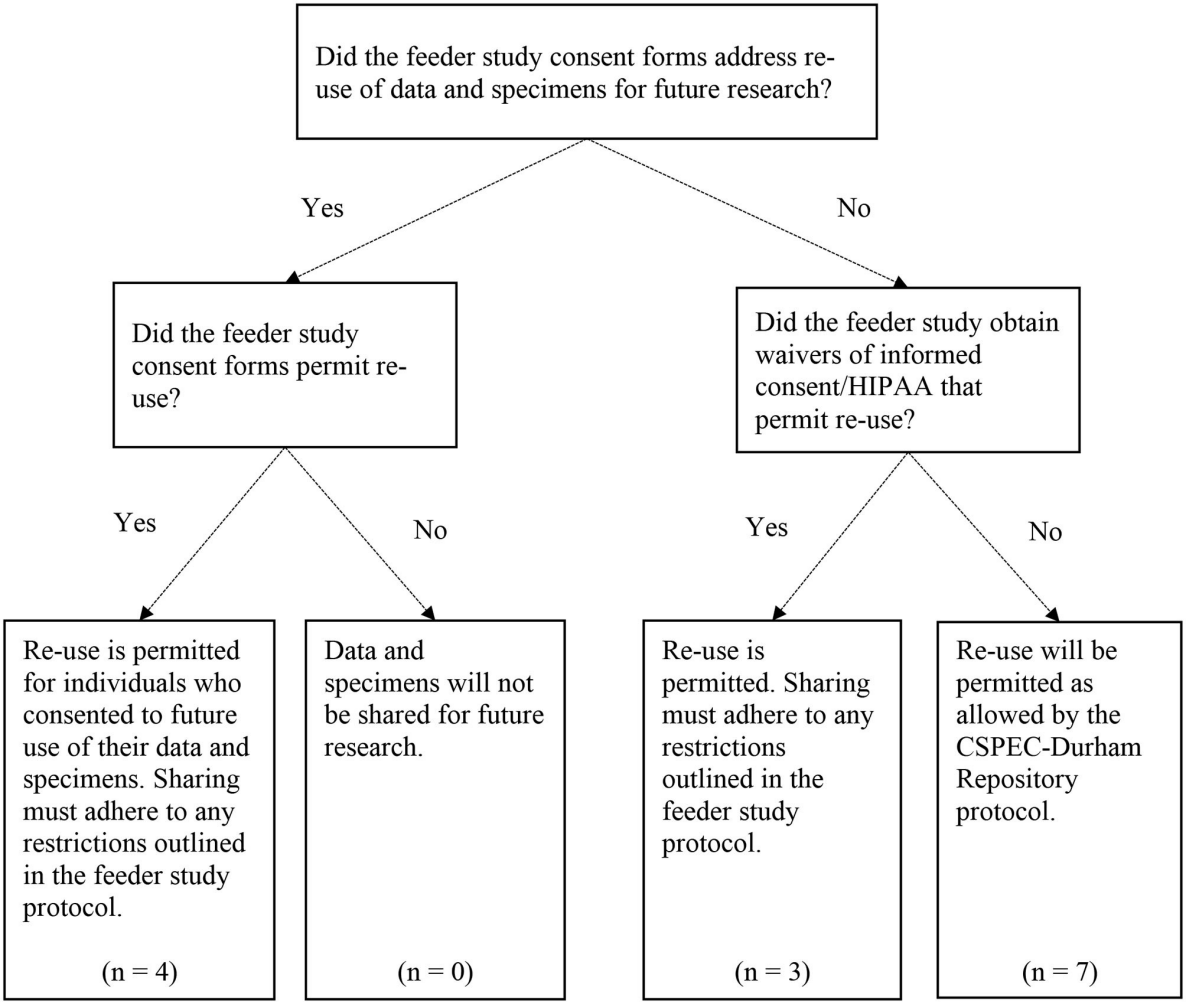
Data and specimen re-use decision points for the CSPEC-Durham Repository. The CSPEC-Durham Repository includes *N* = 15 total feeder studies; however, one feeder study uses administrative data only and does not involve re-use of Veteran data and/or specimens.

We then developed and implemented a formal process for determining whether each study's data and specimens could be shared for future research, and if there were any restrictions on data and specimen sharing (Figure 1). Following guidance from our local IRB, all IRB-approved versions of the study protocol, informed consent form (ICF), Health Insurance Portability and Accountability Act (HIPAA) authorization, ICF waiver, and/or HIPAA waiver were obtained for each feeder study. Two CSPEC-Durham study coordinators reviewed these study documents and documented their findings related to sharing permissions. They recorded whether the study participants had previously consented to the use of their data and specimens for future research. If the participants agreed to future sharing, the reviewers documented any restrictions; e.g., only sharing the data and specimens with researchers within the VA, or for particular research questions (e.g., future research on the causes or treatment of the disease only). The reviewers also noted whether study participants had consented to be re-contacted for future research studies.

Since we included older legacy studies in our repository, some studies did not explicitly address use of the data and specimens for future research in the study consent forms or other documents. For example, a study evaluating the prevalence of colorectal cancer in an average-risk cohort recruited all Veteran participants from 1994 to 1997; the study's ICF was developed prior to the enactment of HIPAA in 1996 (12), and therefore did not include language about the future use of participants' data. Studies in which data and specimen sharing was not explicitly addressed were noted in the review document. The Durham VA IRB approved the inclusion of these legacy studies in our repository and the future sharing of data and specimens if the future research to be conducted was within the scope of the original consent.

We created a study protocol, as well as standard operating procedures, to outline the administration of the repository, types of data and specimens to be shared, data access, methods of data and specimen storage and transfer, and mechanisms for protecting the participants' identities and information. In this protocol, we identified all feeder studies, and categorized each feeder study based on the extent to which it permitted re-use of study data and specimens and/or future re-contact of study participants. In total, we evaluated 15 feeder studies for potential inclusion in our repository, and all 15 studies met our criteria for inclusion, although these studies varied in their restrictions for how data and specimens can be re-used. Of these 15 studies, six were active studies and nine were legacy studies. The CSPEC-Durham Repository protocol was approved by the Durham VA Health Care System IRB in August 2016.

### Database Development and Request Tracking

We designed our repository database to be structured around metadata (i.e., common set of variable descriptors applicable for any study). The metadata-driven architecture is used to manage the data and specimens across all feeder studies and to support the sharing of these data and specimens for future use by approved investigators. While the specific variables differ by study, the metadata across all feeder studies include items such as the dictionary ID, variable label, value description, and data types. As shown in Table 1, using metadata allows us to share the same types of data across feeder studies of diverse topics and designs.

**Table 1:** Common variable descriptors used across feeder studies and examples by feeder study.

**Descriptors**			
Variable Name:	Name of the variable		
Dictionary ID:	Variable ID number		
Variable Label:	A short description of the variable; the variable label only appears when no survey question is available		
Survey Question:	Number and text of survey question from which variable is derived; the survey question only appears when it is available		
Value Descriptions:	Description of possible values for categorical data		
Value Min:	Minimum value possible		
Value Max:	Maximum value possible		
Data Types:	Data types, listed for Generic, SAS, R, SQL, C#, and XML		
Is Nullable:	If true (i.e., is Nullable = 1), a null value is possible		
Form Section:	Section on form or survey from which variable is derived		
Table Name:	Name of database table from which variable is derived		
Variable descriptor^*^	**Colorectal cancer**	**Amyotrophic lateral sclerosis**	**Gulf War research**
Variable name	Colonoscopy	Speech	Act mod days
Dictionary ID	110238	120294	100694
Variable label		Speech	
Survey question	15. Have you ever had a colonoscopy (tube with a light inserted into colon after you are given medicine to make you sleepy)?		18. On how many days did you engage in moderate physical activity (like a brisk walk) in the last 7 days?
Variable description		Please indicate the category that most describes your current state of health: Speech	
Value descriptions	1 = Yes 2 = No	0 = Loss of usual speech 1 = Speech combined with non-vocal communication 2 = Intelligible with repeating 3 = Detectable speech disturbance 4 = Normal speech processes	
Value min			0
Value max			7
Data types	Generic [integer], SAS [4.], R [int], SQL [tinyint], C# [Byte?], XML [xsd:integer]	Generic [integer], SAS [6], R [int], SQL [smallint], C# [Int16?], XML [xsd:integer]	Generic [integer], SAS [4.], R [int], SQL [tinyint], C# [Byte?], XML [va:tinyint]
Is nullable	True	True	True
Form section	Form 01 Clinic Survey Form—Medical History	Veterans ALS Registry Questionnaire—B. ALS Functional Rating Scale	Baseline survey—lifestyle and activities
Table name	AllForm01	ALS Questionnaire	Survey Parent

* *The variable descriptors used are survey item/question dependent and, therefore, some fields are blank for particular variables of each study*.

The common set of variable descriptors are used to generate application code for data entry and validation, creation of data dictionaries, and data extracts used to fulfill specific data sharing requests. The use of metadata was intended to eliminate repetitive and error-prone manual steps, to ensure data provenance, and to create a common structure despite differences in the types of feeder studies. Since requestors typically only need access to a subset of the data collected for a particular feeder study for their own analyses, the use of metadata allows us to create individualized data dictionaries for each requestor and to track all transfers of data to each requestor. We used a similar process to facilitate specimen sharing; common descriptors across feeder study specimens were used to develop specimen-specific applications, inventories, and shipping manifests.

The metadata tables are stored in a relational database and data maintenance history logs, data extract snapshots, and histories of all source code are retained. The repository data are stored on a Microsoft SQL Server behind VA firewalls and access is controlled through active directory security groups for study-specific IRB-approved personnel. Microsoft SQL Server Management Studio is used to work with the data (e.g., data updates, data pulls for sharing). While metadata for the feeder studies are comingled, each feeder study's data are stored in a separate database (with access controlled by the IRB staff list), and there is no comingling of the feeder study data. We adhere to all VA directives about how to securely store and work with Veteran data.

We also created a Research Electronic Data Capture (REDCap) database (13) to track the study documentation for all researchers who submit a formal application to use data and specimens from the CSPEC-Durham Repository. The study documentation includes the requestor's contact information, application materials, and IRB approval letters; evaluations, scores, and recommendations for each request; dates of all study agreements executed; and details and dates of all data and specimen transfers. In addition, the REDCap system is used to track communications with the requestor from the initial inquiry through study completion. The REDCap database is behind VA firewalls and can only be accessed by IRB-approved study staff.

### Data and Specimen Sharing

We developed a comprehensive process for reviewing requests from VA and non-VA researchers to use the data and specimens stored in the CSPEC-Durham Repository. The review process begins when an investigator submits a full application, comprised of a data and specimen request form, documentation of IRB approval, documentation of funding support, and biosketches for all co-investigators and biostatisticians. In the request form, the investigator identifies the feeder study of interest, which variables and/or biosamples are requested, and whether Veterans have been consulted in the study design, among other details.

Following receipt of a full application, we convene the CSPEC-Durham Repository's Scientific and Ethical Oversight Committee (SEOC) to review the request. For each request, the SEOC is comprised of a minimum of two content reviewers (i.e., subject matter experts) who evaluate the proposed study's scientific and ethical merit; at least one statistical reviewer who focuses primarily on the study design, statistical analysis plan, and considerations of the implications of the sample size for the proposed study; and at least one Veteran representative who considers the relevance of the research question to Veterans and the extent to which Veterans have been consulted or engaged in the study design (which is a dedicated section of the application). The Veteran representative is invited from a larger team of Veterans who take turns reviewing each request based on their availability and interest. Each reviewer is asked to independently review all materials, evaluate the request on a series of criteria using a web-based evaluation tool, and provide an overall score of the request that reflects the quality of the application and the prioritization of the specific request. The level of prioritization is particularly critical for specimen requests because there are finite amounts of most specimen types. Once the independent evaluations are completed, the SEOC reviewers and repository administrators hold a review meeting to discuss the reviewers' comments and determine if the request should be approved, approved conditionally with revisions, recommended for resubmission, or declined.

If a request is approved, the CSPEC-Durham Repository team works directly with the requestor and the requestor's institution to execute a data use agreement (DUA) and, if specimens will be used, a material transfer agreement (MTA). Data and specimens will only be shared with approved investigators once these agreements are fully executed to ensure the security of the data and specimens during transfer, storage, and analysis. The agreements specify all terms of the data and/or specimen sharing, including who will have access, methods of transfer and storage, ownership, reporting of results, and destruction or return of the data and/or specimens following study completion. Examples of these terms are presented in Table 2. The requested data and specimens are then securely transferred to the investigator for the approved research study.

**Table 2:** Sample terms of agreement for data use agreements (DUAs) and material transfer agreements (MTAs).

**Topic**	**Terms of agreement**
**DUA terms of agreement**	
General	The Requestor represents that CSP Data will be used solely for the purpose of the Study as specified.
Data ownership	The Requestor is designated as Custodian of the CSP Data provided under this Agreement and does not own the data.
Data management	The Requestor affirms that the requested CSP Data is the minimum necessary to achieve Study goals involving CSP Data.
Unauthorized disclosure	The Requestor shall immediately report any use or disclosure of CSP Data not provided for in this Agreement or any non-compliance with this Agreement to the CSP Center Contact.
Institution approvals	CSP will be provided with written evidence of the IRB determination before release of CSP Data.
Products	The Requestor shall present any product resulting from the CSP Data in aggregated form.
**MTA terms of agreement**	
Research materials	The Research Materials will only be used for research purposes by the Recipient of the Biological Materials in his/her laboratory, for the research project described under suitable containment conditions.
Commercialization	The Human Biological Materials shall not be used for any commercial purposes, including selling, commercial screening, or transfer of the Human Biological Materials to a third party for commercial purposes.
Data management	The Recipient agrees to retain control over this Material and further agrees not to transfer the Material to other people not under his or her direct supervision without advance written approval of the Provider.
Intellectual property	The Recipient acquires no intellectual property as a result of the transfer of the Materials identified under this Agreement.

*CSP, Cooperative Studies Program*.

### Return of Derived Data

Since the objective of the CSPEC-Durham Repository is to support additional research on Veteran health, we require all approved investigators to return the data derived from their analyses to the repository. This includes analytic data derived from the study data and assay data derived from use of the study specimens. We further developed this process in August 2020 by standardizing the requirements related to the return of derived data. These requirements include returning data in a mutually agreed upon timely manner after publication of results, providing a codebook or related documentation that describes any new or collapsed variables in the analytic dataset, and, if specimens were shared, providing an assay protocol that describes how the specimens were stored and analyzed. The returned data can then be made available to other researchers for validation and subsequent analyses.

## Results

The CSPEC-Durham Repository includes Veteran data and specimens from 15 feeder studies with a focus on three primary disease areas: colorectal cancer, amyotrophic lateral sclerosis (ALS), and Gulf War research (Table 3). Seven of these 15 feeder studies relate to these three primary content areas, and each of these seven feeder studies were funded by the VA Cooperative Studies Program (CSP). While we do not currently anticipate requests for the other feeder studies, we included them as a means for long-term storage and security of the previously collected data.

**Table 3:** Data and specimens collected for three primary content areas of the CSPEC-Durham Repository.

**Study title**	**Subjects (*N*)**	**Eligibility criteria**	**Data collected**	**Timing of data collection**	Samples collected^*^	**Timing of specimen collection**
Prospective Evaluation of Risk Factors for Large Colonic Adenomas in Asymptomatic Subjects (CSP #380)	3,121	Veterans ages 50–75 who underwent screening colonoscopies from 1994 to 1997	Results of GI exams, medical history, family history, lifestyle factors, GWAS results	Baseline: 1994–19975-year GI exams10-year GI exams	Blood, tissue	Baseline: 1994–1997Longitudinal: 1994-Present
National Registry of Veterans with Amyotrophic Lateral Sclerosis (CSP #500A)	1,225	Veterans with a verified diagnosis of ALS in 2003–2007, regardless of VA user status	ALS functional rating score, family history, lifestyle factors, use of ventilatory or feeding support, GWAS results	Baseline: 2003–2007Every 6 months for up to 5 years	Blood	Baseline: 2003–2007
Gulf War Era Cohort and Biorepository (CSP #585)	1,274	Veterans who served between July 1990 and August 1991, regardless of deployment or VA user status	Prior exposures if deployed to the Gulf region, family history, physical and mental health, lifestyle factors, GWAS results	Baseline: 2014–2016	Blood	Baseline: 2014–2016

*GI, gastrointestinal; GWAS, genome-wide association study; ALS, amyotrophic lateral sclerosis*.

* *DNA samples have been extracted from the blood samples for each of these feeder studies*.

For the three primary disease areas, the repository contains data and biospecimens such as Veterans' demographic, military service, healthcare utilization, and clinical data, as well as tissue and blood samples. The data and specimens were collected longitudinally at multiple time points for the first two of these three disease areas, allowing for research on disease progression and how risk factors differentially affect clinical and survival outcomes, and cross-sectionally for the third disease area. In each of these cases, the research data and specimens can be linked with the participants' VA medical records to assess longer-term clinical and survival outcomes. The ability to link the feeder study specimens with rich clinical and research data provides opportunities for genetic and molecular association analyses to inform Veteran care.

### Colorectal Cancer

Asymptomatic Veterans aged 50–75 years were enrolled in the study, “Prospective Evaluation of Risk Factors for Large Colonic Adenomas in Asymptomatic Subjects,” (CSP #380) between 1994 and 1997 at 13 geographically diverse VA medical centers (14). Each of the 3,121 study participants underwent a baseline screening colonoscopy as part of the study and were followed for 10 years or until death. The cohort's clinical outcomes, including prevalence of advanced colorectal neoplasia and colorectal cancer, at the time of the study (14), after 5 years (15), and after 10 years (16) were previously reported.

The study data stored in the repository includes the results of the baseline colonoscopies as well as other gastrointestinal (GI) exams completed during the longitudinal follow-up period. Sixty-one percent (*N* = 1,915) of this cohort had at least one surveillance colonoscopy within 10 years of their baseline exam (16). Survey data, including medical history, family history, and lifestyle factors, such as tobacco use, alcohol use, physical activity, and diet, are also stored.

The specimen repository includes colorectal tissues biopsied during colonoscopies and other GI exams completed as part of the study and as part of routine clinical care. These formalin-fixed paraffin-embedded (FFPE) and Bouin's-fixed tissue samples are stored in VA pathology labs until they are ready to be discarded or used for future research according to VA policy. The study team works with the local sites to retrieve these tissue samples and have them transferred to the Southern Arizona VA Healthcare System (SAVAHCS) in Tucson, Arizona for long-term storage. Tissue samples from some local sites may be stored temporarily at the Durham VA Health Care System for coding purposes. To date, more than 1,800 of these tissues have been added to the specimen repository governed by the CSPEC-Durham Repository and physically located at SAVAHCS. Additional tissues will be retrieved and added to the repository over time as the tissues become available for research purposes. DNA will be extracted from these tissue samples and made available in the repository as well.

The repository also includes frozen blood and tissue samples collected from 815 study participants during their baseline colonoscopy exams, and DNA extracted from these samples. Serum and lymphocytes were collected from those participants with a large polyp (i.e., at least 1 cm); serum and lymphocytes were also collected from age- and sex-matched participants with no polyps detected. Normal-appearing tissue samples and polyp tissues were biopsied from these participants and stored for future use. Each of these cross-sectionally collected specimens have been frozen since baseline and are currently stored at the Massachusetts Veterans Epidemiology Research and Information Center (MAVERIC) in Boston, Massachusetts. A genome-wide association study (GWAS) of these DNA samples has been conducted (17), and the results will be made available through the repository.

### Amyotrophic Lateral Sclerosis

The “National Registry of Veterans with Amyotrophic Lateral Sclerosis” (CSP #500A) enrolled 2,068 Veterans with an ALS diagnosis between April 2003 and September 2007 (18). Each ALS diagnosis was confirmed by a neurologist, providing information on the type of ALS diagnosis, site of onset, and date of diagnosis. Participants, who were recruited from all 50 states, self-reported their symptoms and the severity of their symptoms through phone interviews at baseline and every 6 months for up to 5 years. The ALS Functional Rating Score was used to monitor their health and functional status over time. Additional survey data included in the repository include family history, smoking status, medications, comorbidities, surgical history, and use of ventilatory or feeding support.

Each participant in the ALS Registry was asked to provide a DNA sample to be included in the study's DNA Bank for future research. More than half of the participants (*N* = 1,168) provided a DNA sample, most commonly by a blood sample (85% vs. 15% with a saliva sample) (18). These cross-sectional blood and DNA samples are all governed by our repository, physically stored at MAVERIC, and can be used for future research on ALS causes and treatment. The repository also contains the results of a GWAS of ALS diagnosis and survival using these samples (19).

### Gulf War Research

Veterans who served during the 1990–1991 Gulf War era were enrolled in the “Gulf War Era Cohort and Biorepository” (GWECB, also referred to as CSP #585) between 2014 and 2016 (20). The goal of the GWECB was to collect data to be used for future research on diverse health concerns specific to this cohort of Veterans, including Gulf War Illness (Table 4). A total of 1,344 Veterans were enrolled in the GWECB, including 1,275 for whom we have survey data, health records, and a blood sample (i.e., fully enrolled) and 69 for whom we have surveys and health records. The GWECB sample reflected the geographic distribution of Veterans across the four U.S. Census regions and included Gulf War era Veterans regardless of their deployment, health status, or use of VA healthcare.

**Table 4:** Glossary of terms.

**Term**	**Definition**
Active study	Feeder study in which collection and/or analysis of the data/specimens is occurring currently by the study team
Data use agreement	A legal document describing the terms of agreement for the transfer and use of data between the institution providing the data and the institution/investigator requesting to use the data for research purposes
Feeder study	An individual research study with an IRB-approved protocol for which the collected data and/or specimens are stored and available for sharing through our CSPEC-Durham Data and Specimen Repository
Gulf War Illness	Chronic, multi-symptom health condition affecting Veterans who served in the 1990–1991 Gulf War that is not explained by other medical diagnoses or standard laboratory tests. Common symptoms include fatigue, cognitive impairment, chronic pain, sleep problems, gastrointestinal issues, and skin problems. Multiple diagnostic definitions are used to identify cases of Gulf War Illness (21, 22)
Legacy study	Feeder study in which the collection and analyses of data/specimens have been completed by the study team
Material transfer agreement	A legal document describing the terms of agreement for the transfer and use of human biological specimens between the institution providing the specimens and the institution/investigator requesting to use the specimens for research purposes
Metadata	Common set of variable descriptors (e.g., IDs, variable labels, value descriptions, etc.) for data and specimens collected across feeder studies that is used to structure the repository database

The cross-sectional survey data in the repository includes prior exposures during military service if deployed to the Gulf region, family history, physical and mental health, including the severity, frequency, and functional impact of specific conditions, and lifestyle factors, such as physical activity, tobacco use, and alcohol use. The participants also consented to be re-contacted by the GWECB team for possible participation in future studies.

The repository also includes plasma and buffy coat samples, as well as extracted DNA, for each study participant. These samples were collected at baseline. Our repository governs these specimens, which are physically stored at MAVERIC. A GWAS using these DNA samples has been conducted. The analysis of the GWAS data is in progress; the GWECB team plans to publish the results and make them available for sharing through the repository in the future. In addition, an algorithm is being developed by the GWECB team that will help to identify cases of Gulf War Illness in this cohort using the self-reported survey data; manuscripts describing the results and the methodology are forthcoming.

### Data and Specimen Requests

As of December 2020, we have received 10 formal applications to use data and/or specimens from the CSPEC-Durham Repository. Seven of the 10 applications were approved for data and/or specimen sharing following the scientific and ethical review process. Of these 7 approved requests, 3 requests have been fulfilled, 2 requests have a fully executed DUA/MTA but the data/specimens have not yet been transferred, and 2 requests have a DUA/MTA in progress and the data/specimens will be transferred thereafter. The amount of time required to review each request, execute the DUA/MTA, and transfer the data/specimens has varied by request due to the complexity of each request, review and negotiations of all legal agreements, and other factors.

## Discussion

Through the development of the CSPEC-Durham Repository, we created a mechanism for facilitating future research on diverse health topics affecting Veterans. This multi-study repository provides a single structure that can be used to support the sharing of data and specimens across multiple content areas, for different types of research studies (e.g., active vs. legacy studies, cross-sectional vs. longitudinal data collection, etc.), and across types of data (e.g., survey data, medical record data, genomic data) and specimens (e.g., blood, tissue, DNA) collected. This resource can support additional research, including genetic and molecular association analyses, aimed to better understand, diagnose, and treat chronic diseases affecting Veterans and the general population, which closely aligns with current ORD, VA, and national research priorities.

The CSPEC-Durham Repository adds to the growing number of data repositories and biorepositories within the VA, reflecting the high prioritization of research collaboration to improve care delivery. The VA's Million Veteran Program (MVP) has enrolled more than 825,000 Veterans since 2011 in order to facilitate research assessing genetic influences on health and disease to develop precision medicine (23, 24). The Veterans Precision Oncology Data Commons similarly aims to support research in precision oncology through the sharing of clinical and genomic data available for cancer patients in the VA (25). Other examples of repositories in the VA focused on specific topic areas include the Mental Illness Research Education and Clinical Center (MIRECC) (26) and the VA Biorepository Brain Bank for ALS research (27). The CSPEC-Durham Repository is unique in that it is a center-wide repository, not specific to a single health topic, and allows for data and specimen sharing across legacy studies for which data and specimen sharing would otherwise not be possible.

One of the strengths of our repository is the metadata-driven database architecture, which has automated steps across the data life cycle, including data entry and data extraction for approved investigators. We also successfully applied this metadata-driven approach to the specimens stored in our repository. This approach has increased efficiency from a repository management perspective and allowed for improved safeguarding of the study data and specimens. Another asset has been the inclusion of legacy studies in the repository. Given that Veteran participants of these studies provided their time and efforts to research on particular health issues, it is important to be able to use the information and specimens they shared to advance research and innovations in these areas (within the scope of their original consent). Including these studies reflects the trend over time toward increased transparency and access to research data.

There are also some limitations. The number of participants across the repository feeder studies is relatively small when compared to biobanks such as MVP (23, 24). For this reason, researchers requesting the data are asked to provide their statistical plan and reflect on the implications of the sample size for their particular study. The merits of their statistical plan and plans to address any data limitations are reviewed and evaluated by one or more statistical reviewers on the SEOC as part of the review process. In addition, now that we have developed the repository structure and database architecture, we have a well-established mechanism to adopt additional feeder studies, including those that may be actively recruiting participants. This may help to increase the number of study participants for whom we have data and specimens available for each content area. A second limitation is that the data and specimens for the GWECB were collected at a single point in time, and the specimens collected from ALS Registry participants were also cross-sectional. However, the participants from the GWECB consented to be re-contacted for additional studies related to Gulf War research, which may allow for collection of data at subsequent time points. In addition, while the specimens were collected at a single point in time from the ALS Registry participants, their surveys were completed at multiple time points.

Our repository team has taken steps to integrate our resources with other repository initiatives within the VA to increase efficiencies for our staff and researchers alike and to improve visibility of our resources. As one key example, our team works closely with the Integrated Veteran Epidemiologic Study Data Resource (INVESTD-R) team, which has created a publicly available web-based tool to describe the resources available for continued research within the VA CSP (28). The feeder studies included in the CSPEC-Durham Repository are highlighted on this resource, allowing us to potentially reach more diverse researchers and consolidate our resources within the context of the larger CSP research program. We continue to work with the INVESTD-R team to streamline review processes and other documentation for researchers requesting data and specimens across the VA CSP. In addition, following existing models within the VA, all study specimens in our repository are physically stored at approved VA biorepositories. While our team governs all aspects of data management for these specimens, including the request process, crosswalk between the specimens and the corresponding study data, and the sharing of specimens with approved researchers, the laboratory personnel at the VA biorepositories ensure secure storage and maintenance of the physical specimens. These collaborations help to leverage our respective areas of expertise and available resources to best support continued Veteran health research. Within the larger VA ORD, there are ongoing discussions across the program about how to further integrate existing repository resources while still adhering to all VA data sharing requirements and adhering to the permissions documented in the original consent forms.

There are continued opportunities to advance Veteran health research and delivery of care through collaboration with other VA repositories. As one example, we hope to create a streamlined review process for requests to use ALS specimens with the VA Biorepository Brain Bank, which stores central nervous system (CNS) tissues for Veterans with ALS. Creating a joint process will allow interested investigators to simultaneously request DNA samples from our repository and tissue samples from the Brain Bank for the same individuals. Furthermore, there is opportunity to link our GWECB with additional Gulf War research resources in the VA. These collaborative activities can create further efficiencies in the storage and sharing of Veteran data and specimens, with the overarching goal of sharing VA data nationally and using this information to improve the health and care of Veterans.

## Data Availability Statement

The original contributions generated in the study are included in the article. Details regarding the data and specimens included in the CSPEC-Durham Data and Specimen Repository are available on the INVESTD-R website (https://www.vacsp.research.va.gov/CSPEC/Studies/INVESTD-R/Main.asp). Inquiries about requesting to use the data and specimens can be directed to the corresponding authors.

## Ethics Statement

The feeder studies included in the CSPEC-Durham Data and Specimen Repository involving human participants were reviewed and approved by the Durham VA Health Care System Institutional Review Board and/or the VA Central Institutional Review Board. Written informed consent for participation was not required for this study in accordance with the national legislation and the institutional requirements.

## Disclosure

The views expressed in this article are those of the authors and do not necessarily reflect the position or policy of the Department of Veterans Affairs, the VA Cooperative Studies Program, or the U.S. government.

## Author Contributions

MO'L: conceptualization, methodology, project administration, writing, and revising the manuscript. RLW and CW: conceptualization, methodology, and revising the manuscript. AP: conceptualization, methodology, project administration, and revising the manuscript. DP and KS: conceptualization, methodology, revising the manuscript, and supervision. BC, RJ, and TR: methodology and revising the manuscript. All authors: contributed to the article and approved the submitted version.

## Conflict of Interest

The authors declare that the research was conducted in the absence of any commercial or financial relationships that could be construed as a potential conflict of interest.
